# Progesterone receptor membrane component 1 reduces cardiac steatosis and lipotoxicity via activation of fatty acid oxidation and mitochondrial respiration

**DOI:** 10.1038/s41598-021-88251-2

**Published:** 2021-04-22

**Authors:** Sang R. Lee, Jun H. Heo, Seong Lae Jo, Globinna Kim, Su Jung Kim, Hyun Ju Yoo, Kyu-Pil Lee, Hyo-Jung Kwun, Hyun-Jin Shin, In-Jeoung Baek, Eui-Ju Hong

**Affiliations:** 1 College of Veterinary Medicine, Chungnam National University, 99 Daehak-ro, Suite 401 Veterinary medicine Bldg., Yuseong, Daejeon, 34134 Korea; 2 Department of Convergence Medicine, University of Ulsan College of Medicine, Asan Medical Center, Seoul, 05505 Republic of Korea

**Keywords:** Biochemistry, Lipids, Endocrinology, Endocrine system and metabolic diseases

## Abstract

Obesity is implicated in cardiovascular disease and heart failure. When fatty acids are transported to and not adequately oxidized in cardiac cells, they accumulate, causing lipotoxicity in the heart. Since hepatic progesterone receptor membrane component 1 (*Pgrmc1*) suppressed de novo lipogenesis in a previous study, it was questioned whether cardiac *Pgrmc1* protects against lipotoxicity. Hence, we focused on the role of cardiac *Pgrmc1* in basal (Resting), glucose-dominant (Refed) and lipid-dominant high-fat diet (HFD) conditions. *Pgrmc1* KO mice showed high FFA levels and low glucose levels compared to wild-type (WT) mice. *Pgrmc1* KO mice presented low number of mitochondrial DNA copies in heart, and it was concomitantly observed with low expression of TCA cycle genes and oxidative phosphorylation genes. *Pgrmc1* absence in heart presented low fatty acid oxidation activity in all conditions, but the production of acetyl-CoA and ATP was in pronounced suppression only in HFD condition. Furthermore, HFD *Pgrmc1* KO mice resulted in high cardiac fatty acyl-CoA levels and TG level. Accordingly, HFD *Pgrmc1* KO mice were prone to cardiac lipotoxicity, featuring high levels in markers of inflammation, endoplasmic reticulum stress, oxidative stress, fibrosis, and heart failure. In vitro study, it was also confirmed that *Pgrmc1* enhances rates of mitochondrial respiration and fatty acid oxidation. This study is clinically important because mitochondrial defects in *Pgrmc1* KO mice hearts represent the late phase of cardiac failure.

## Introduction

The incidence of heart disease is increasing in modern society. With dietary factors being strongly linked to life-threatening heart diseases^1,2^, abnormal metabolism should be considered a trigger of cardiovascular conditions. Obesity is a fundamental disorder that is closely related to the pathogenesis of metabolic diseases. It promotes development of insulin resistance and an increase in free fatty acids (FFA)^3^ because of increased fat mass, decreased fatty acid disposal, and acquired insulin resistance^4^. Some clinical studies have implied that characteristics of obesity, including increased fat mass, FFA, and insulin resistance, are associated with cardiac lipid accumulation and coronary heart disease^5,6^.

Energy metabolism in the heart can be greatly affected by excess lipids under conditions of obesity, and it is important to note that the heart primarily relies on fatty acid oxidation for energy production. Fatty acids derived from nutrient sources or generated by lipolysis from adipose tissue enter the heart, and are either used for energy production processes or stored for later use. For energy production, fatty acyl-CoA enters the mitochondria to carry out fatty acid oxidation. Thus, it is converted into acetyl-CoA by oxidation, which is then metabolized in the course of the TCA cycle. NADH and FADH produced by a series of metabolic reactions in the TCA cycle serve to generate ATP via mitochondrial oxidative phosphorylation^7^. However, fatty acyl-CoA can be stored by conversion into other forms of cardiac lipids, including ceramides, diacylglycerols (DAG), and triglycerides (TG). When storage outweighs oxidation, accumulation of ceramides, DAG, and TG can lead to cardiac pathogenesis. While TG is less toxic, DAG and ceramides are the major toxic compounds that affect the heart^8^.

Previous studies have shown that progesterone receptor membrane component 1 (Pgrmc1) suppresses fatty liver development and promotes pancreatic insulin secretion^9,10^. Although the role of Pgrmc1 has been studied in terms of various metabolic functions, including gluconeogenesis and cholesterol synthesis^11,12^, it has not yet been determined whether it is involved in cardiac metabolism. Based on the previous study involving the activation of P450 proteins of PGRMC1^13^, the present study sought to investigate the relationship between Pgrmc1 expression and cardiac pathogenesis including dysfunction of mitochondrial metabolism. This study will firstly report the role of cardiac PGRMC1 protein. In vivo model using *Pgrmc1* whole-body knockout (KO) mice, *Pgrmc1* depletion presented a low expression of mitochondrial DNA (mtDNA) and suppressed fatty acid oxidation activity in the heart. This phenotype of *Pgrmc1* KO mice resulted in low cardiac acetyl-CoA and ATP production when the diet was high in fat (HFD). Furthermore, lipid accumulation resulted in cardiac lipotoxicity upon *Pgrmc1* loss. We compared three different feeding conditions to define the metabolic role of cardiac *Pgrmc1* in animal models and confirmed the role of cardiac-specific *Pgrmc1 *in vitro conditions.

## Results

### *Pgrmc1* absence resulted in a low number of cardiac mtDNA copies and suppressed cardiac mitochondrial metabolism, including TCA cycle, OXPHOS, and fatty acid oxidation

**Figure 1:**
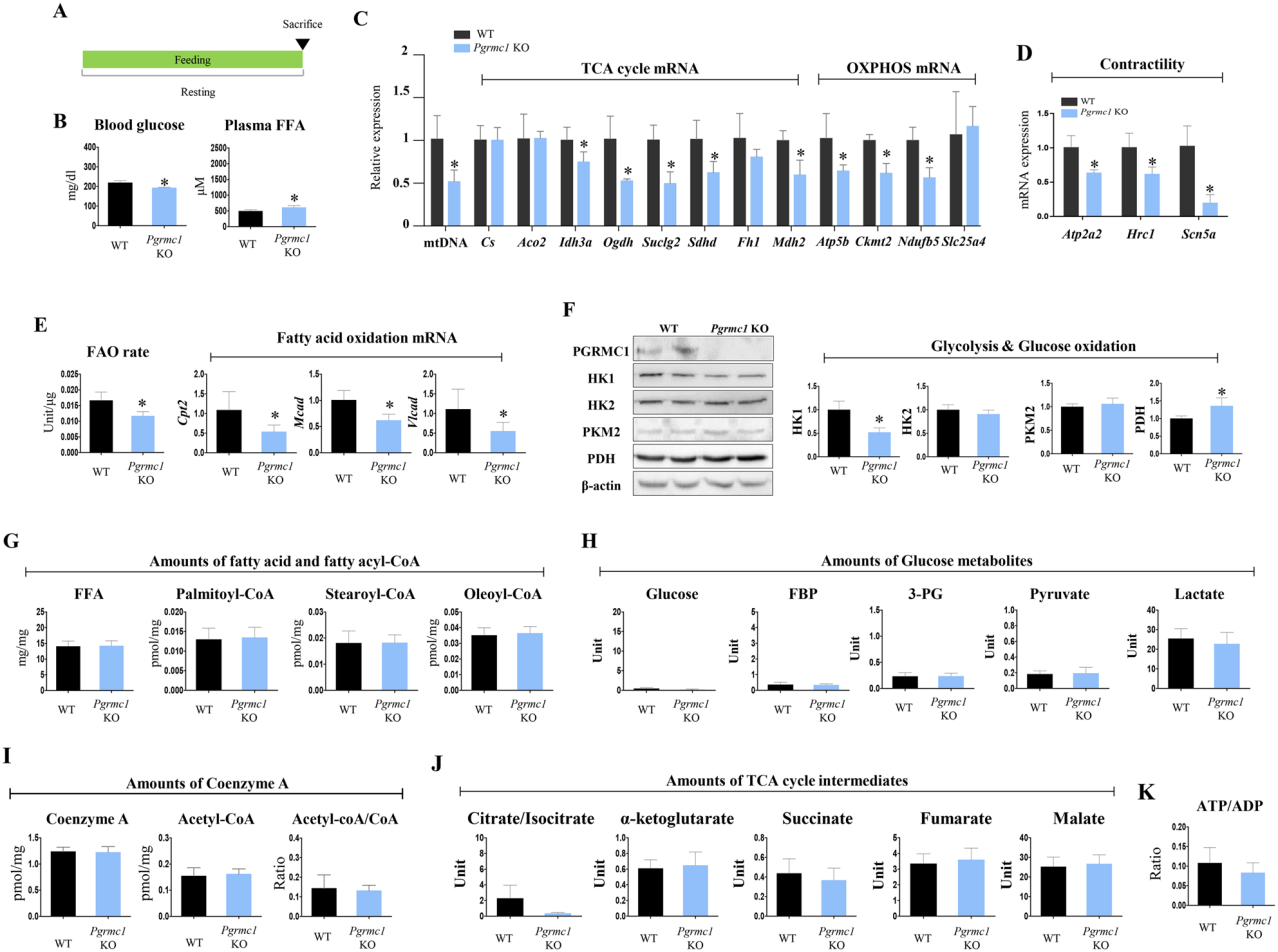
*Pgrmc1* KO mice show low cardiac mitochondrial metabolism in resting state. (**A**) Schematic diagram which presents experimental schedule for resting state. (**B**) Levels of blood glucose (mg/dL) and plasma FFA (µM) in resting WT and *Pgrmc1* KO mice. (**C**) Expression of mitochondrial DNA (mtDNA) in hearts of resting WT and *Pgrmc1* KO mice. Nuclear DNA was used for an internal control. mRNA expression of TCA cycle and OXPHOS in hearts of resting WT and *Pgrmc1* KO mice. *Rplp0* was used for an internal control. (**D**) mRNA expression of contractility markers in hearts of resting WT and *Pgrmc1* KO mice. *Rplp0* was used for an internal control. (**E**) Fatty acid oxidation activity in hearts of resting WT and *Pgrmc1* KO mice. mRNA expression of fatty acid oxidation genes in hearts of resting WT and *Pgrmc1* KO mice. *Rplp0* was used for an internal control. (**F**) Western blot analysis and quantification of glycolysis and glucose oxidation genes in hearts of resting WT and *Pgrmc1* KO mice. β-actin was used for an internal control. (**G**) Cardiac FFA level (µM) and fatty acyl-CoA levels (pmol/mg) in resting WT and *Pgrmc1* KO mice. (**H**) Levels of glucose metabolites (unit) in resting WT and *Pgrmc1* KO mice. (**I**) Levels of coenzyme A (CoA) and acetyl-CoA in resting WT and *Pgrmc1* KO mice. (**J**) Levels of TCA cycle intermediates in resting WT and *Pgrmc1* KO mice. (**K**) Ratio of ATP per ADP in resting WT and *Pgrmc1* KO mice. Values represent means ± SD. *p < 0.05. Student’s t test was performed. Total numbers of mice used for experiment were 5 (control WT), 4 (control *Pgrmc1* KO).

In resting condition when mice were fed ad libitum (Fig. 1A), levels of blood glucose were decreased (p < 0.05, 88.1% vs. WT) while levels of plasma FFA were increased (p < 0.05, 1.23-fold vs. WT) in *Pgrmc1* KO mice (Fig. 1B). Interestingly, the expression of mtDNA was lower (p < 0.05, 51.2% vs. WT) in the *Pgrmc1* KO hearts, suggesting the fundamental defect of cardiac mitochondria (Fig. 1C). Consistently, *Pgrmc1* KO hearts significantly suppressed cardiac mRNA expression of TCA cycle genes, *Idh3a*, *Ogdh*, *Suclg2*, *Sdhd*, and *Mdh2* (p < 0.05, 74.6, 52.3, 49.5, 61.5, and 59.9%, respectively), and oxidative phosphorylation (OXPHOS) genes, *Atp5b*, *Ckmt2*, and *Ndufb5* (p < 0.05, 63, 61.7, and 56.4%, respectively), compared to WT hearts (Fig. 1C). These low energy mediated steps were reflected by suppressed (p < 0.05, 63.4, 61.4, and 19.4%, respectively, vs. WT) expression of contractile genes, *Atp2a2*, *Hrc1*, and *Scn5a*, in the hearts of *Pgrmc1* KO mice (Fig. 1D). Using enzyme-dependent fatty acid oxidation measurement, fatty acid oxidation activity of heart samples, not in vivo fatty acid oxidation rate, was analyzed. As a primary cardiac mitochondrial metabolism, fatty acid oxidation activity was suppressed (p < 0.05, 70.3% vs. WT) in the hearts of *Pgrmc1* KO mice (Fig. 1E). The mRNA expression of *Cpt2*, *Mcad*, and *Vlcad* was also suppressed (p < 0.05, 49.5, 61.4, 49.7%, respectively, vs. WT) in the hearts of *Pgrmc1* KO mice (Fig. 1E). In western blot, we observed the presence of cardiac PGRMC1 (Fig. 1F). While protein expression of hexokinase (HK1) was decreased (p < 0.05, 52.2% vs. WT), protein expression of pyruvate dehydrogenase (PDH) was increased (p < 0.05, 1.36-fold vs. WT) in the hearts of *Pgrmc1* KO mice (Fig. 1F). However, hexokinase2 (HK2) and pyruvate kinase M2 (PKM2) protein expression did not show a significant difference (Fig. 1F). Despite the expression of mitochondrial metabolic genes and fatty acid oxidation activity in *Pgrmc1* KO heart were suppressed, the amount of cardiac metabolites was not changed (Fig. 1G–K).

**Figure 2:**
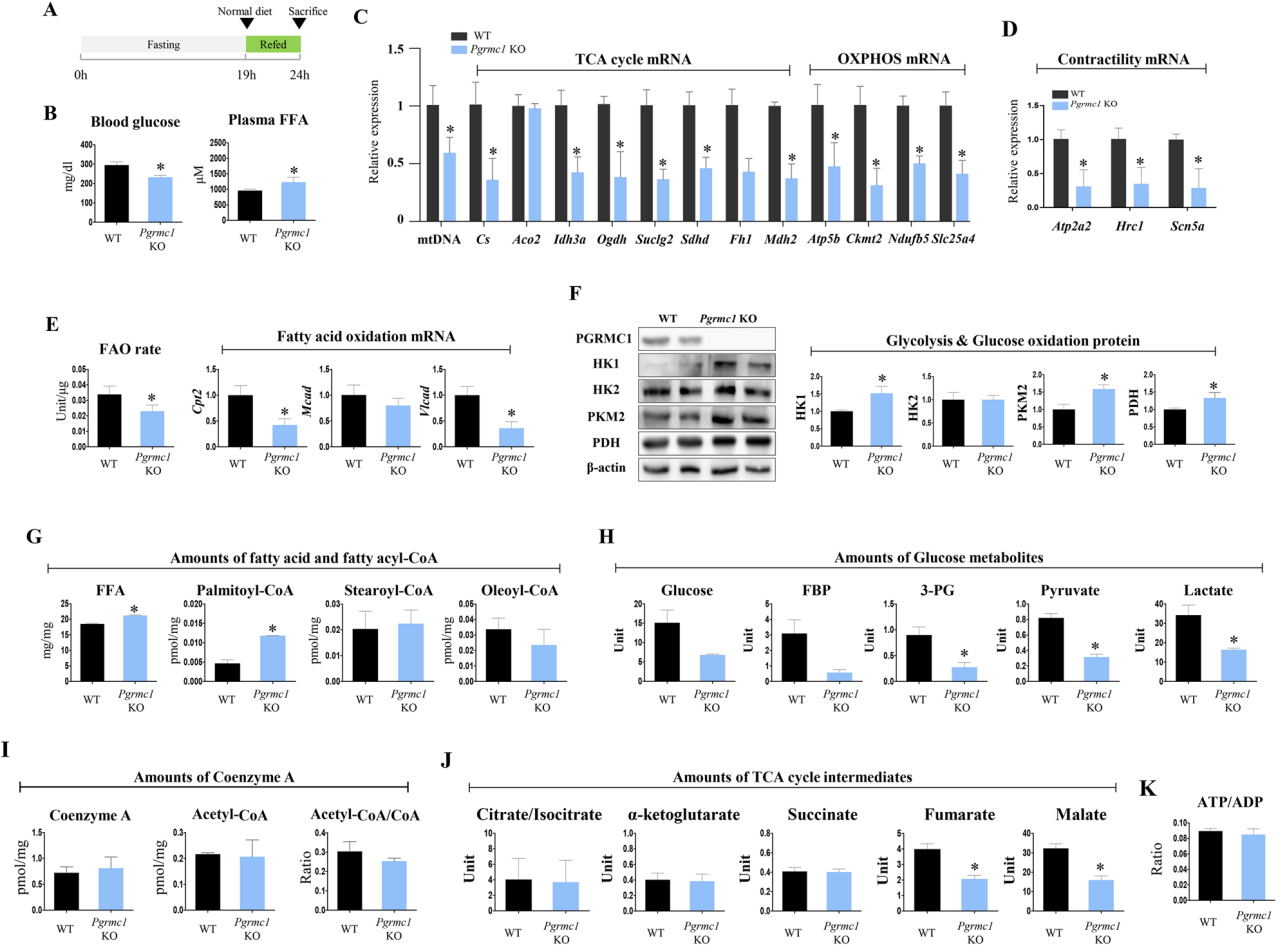
*Pgrmc1* KO mice show high levels of cardiac FFA and paltimoyl-CoA, but low levels of cardiac glucose metabolites and TCA cycle intermediates in refed state. (**A**) Schematic diagram which presents experimental schedule for refed state. (**B**) Levels of blood glucose (mg/dl) and plasma FFA (µM) in refed WT and *Pgrmc1* KO mice. (**C**) Expression of mitochondrial DNA (mtDNA) in hearts of refed WT and *Pgrmc1* KO mice. Nuclear DNA was used for an internal control. mRNA expression of TCA cycle and OXPHOS in hearts of refed WT and *Pgrmc1* KO mice. *Rplp0* was used for an internal control. (**D**) mRNA expression of contractility markers in hearts of refed WT and *Pgrmc1* KO mice. *Rplp0* was used for an internal control. (**E**) Fatty acid oxidation activity in hearts of refed WT and *Pgrmc1* KO mice. mRNA expression of fatty acid oxidation genes in hearts of refed WT and *Pgrmc1* KO mice. *Rplp0* was used for an internal control. (**F**) Western blot analysis and quantification of glycolysis and glucose oxidation genes in hearts of refed WT and *Pgrmc1* KO mice. β-Actin was used for an internal control. (**G**) Cardiac FFA level (µM) and fatty acyl-CoA levels (pmol/mg) in refed WT and *Pgrmc1* KO mice. (**H**) Levels of glucose metabolites (unit) in refed WT and *Pgrmc1* KO mice. (**I**) Levels of coenzyme A (CoA) and acetyl-CoA in refed WT and *Pgrmc1* KO mice. (**J**) Levels of TCA cycle intermediates in refed WT and *Pgrmc1* KO mice. (**K**) Ratio of ATP per ADP in refed WT and *Pgrmc1* KO mice. Values represent means ± SD. *p < 0.05. Student’s t test was performed. Total numbers of mice used for experiment were 8 (refed WT), and 6 (refed *Pgrmc1* KO).

To investigate cardiac metabolism in *Pgrmc1* KO mice according to defined nutritional conditions, we induced a refed state, as illustrated in Fig. 2A. Both blood glucose levels (295 and 232.3 mg/dL; refed WT and *Pgrmc1* KO mice, respectively) and plasma FFA levels (957.6 and 1229.5 μM; refed WT and *Pgrmc1* KO mice, respectively) were enormously high under refed conditions due to sudden feeding after prolonged fasting (Fig. 2B). Under refed conditions, blood glucose levels were significantly decreased (p < 0.05, 78.8% vs. refed WT), while plasma FFA levels were increased (p < 0.05, 1.28-fold vs. refed WT) in refed *Pgrmc1* KO mice (Fig. 2B). Consistent with resting condition, the expression of mtDNA was lower (p < 0.05, 58.8% vs. refed WT) in the hearts of refed *Pgrmc1* KO mice (Fig. 2C).

Suppression in mRNA expression of TCA cycle genes, *Cs*, *Idh3a*, *Ogdh*, *Suclg2*, *Sdhd*, and *Mdh2* (p < 0.05, 35.5, 42.2, 37.9, 36.4, 45.7, and 37.3%, respectively, vs. refed WT), and OXPHOS genes, *Atp5b*, *Ckmt2*, *Ndufb5*, and *Slc25a4* (p < 0.05, 47.2, 31, 50.2, and 41.2%, respectively, vs. refed WT), were still observed in the hearts of refed *Pgrmc1* KO mice (Fig. 2C). Contractility markers, *Atp2a2*, *Hrc1*, and *Scn5a*, also showed low (p < 0.05, 30.7, 34.7, and 29%, respectively, vs. refed WT) mRNA expression in refed *Pgrmc1* KO hearts (Fig. 2D). Fatty acid oxidation activity was significantly lower (p < 0.05, 68.1% vs. refed WT) in the hearts of refed *Pgrmc1* KO hearts (Fig. 2E). The mRNA expression of fatty acid oxidation genes, *Cpt2* and *Vlcad*, was suppressed (p < 0.05, 42.3 and 36.1%, respectively, vs. refed WT) in the hearts of refed *Pgrmc1* KO mice (Fig. 2E). Protein expression of glycolysis genes, HK1 and PKM2, was increased (p < 0.05, 1.52- and 1.59-fold, respectively, vs. refed WT) in the hearts of refed *Pgrmc1* KO mice (Fig. 2F). Protein expression of the glucose oxidation gene PDH was increased (p < 0.05, 1.33-fold vs. refed WT) in the hearts of refed *Pgrmc1* KO mice (Fig. 2F).

Enrichment of glucose and fatty acid resulted in metabolite level changes according to metabolic gene alteration of Pgrmc1 KO hearts. Levels of FFA and palmitoyl-CoA (C16:0) in the hearts of refed *Pgrmc1* KO mice were higher (p < 0.05, 1.15- and 2.53-fold, respectively) than those of WT mice (Fig. 2G). Conversely, levels of fructose-1,6-bisphosphate (FBP), 3-phosphoglycerate (3-PG), pyruvate, and lactate were lower (p < 0.05, 1.15- and 2.53-fold, respectively, vs. refed WT) in the hearts of refed *Pgrmc1* KO mice (Fig. 2H). While the acetyl-CoA level was not changed, fumarate and malate levels were lower (p < 0.05, 51.8 and 49.4%, respectively, vs. refed WT) in the hearts of refed *Pgrmc1* KO mice (Fig. 2I–J). Nonetheless, ATP production was not suppressed in *Pgrmc1* KO hearts (Fig. 2K).

### *Pgrmc1* absence possesses high cardiac fatty acyl-CoAs, but produce low cardiac TCA metabolites, acetyl-CoA, and ATP in HFD

**Figure 3:**
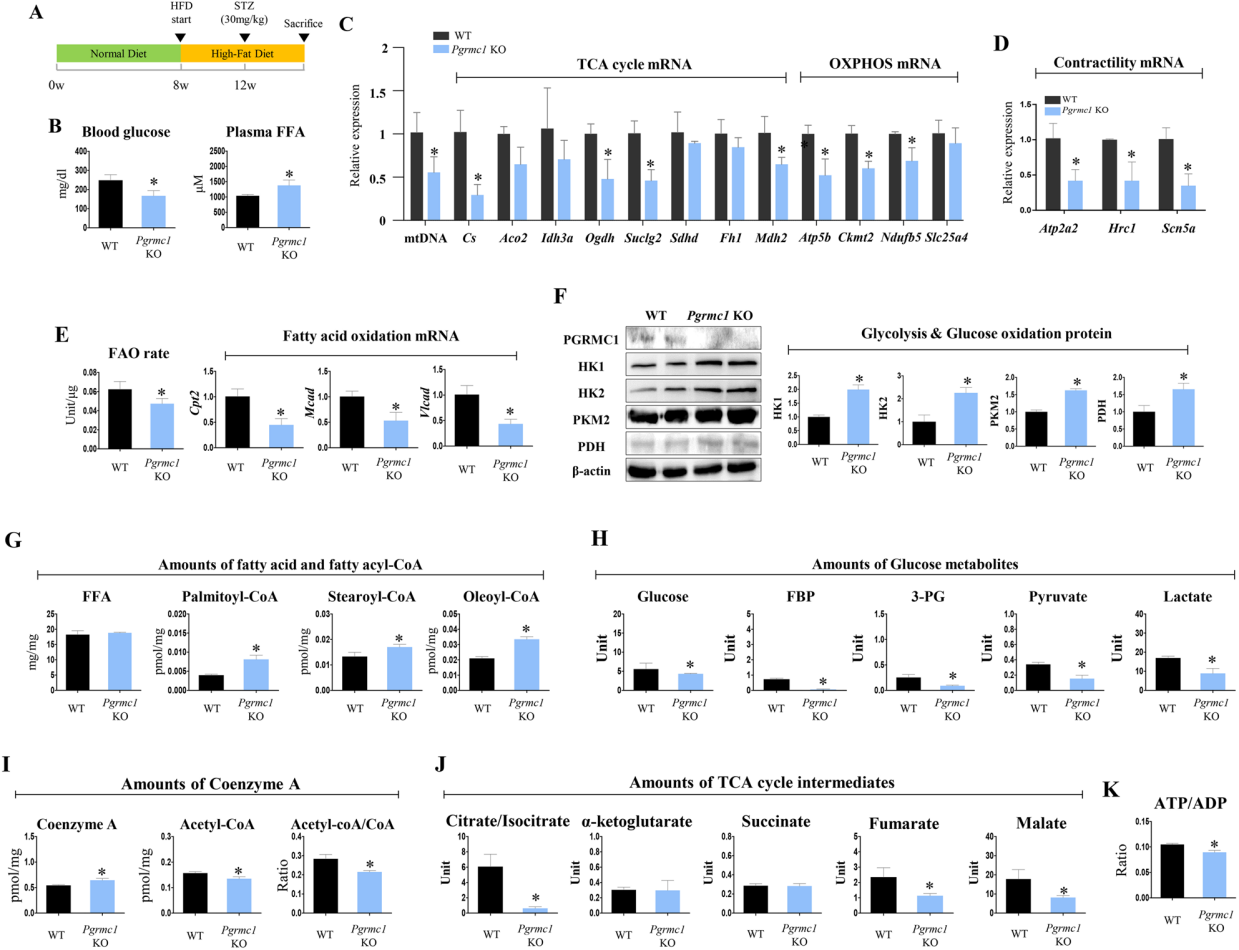
*Pgrmc1* KO mice increases cardiac fatty acyl-CoA levels, but suppresses production of acetyl-CoA and ATP. (**A**) Schematic diagram which presents experimental schedule for HFD state. (B) Levels of blood glucose (mg/dl) and plasma FFA (µM) in HFD WT and *Pgrmc1* KO mice. (**C**) Expression of mitochondrial DNA (mtDNA) in hearts of HFD WT and *Pgrmc1* KO mice. Nuclear DNA was used for an internal control. mRNA expression of TCA cycle and OXPHOS in hearts of HFD WT and *Pgrmc1* KO mice. *Rplp0* was used for an internal control. (**D**) mRNA expression of contractility markers in hearts of HFD WT and *Pgrmc1* KO mice. *Rplp0* was used for an internal control. (**E**) Fatty acid oxidation activity in hearts of HFD WT and *Pgrmc1* KO mice. mRNA expression of fatty acid oxidation genes in hearts of HFD WT and *Pgrmc1* KO mice. *Rplp0* was used for an internal control. (**F**) Western blot analysis and quantification of glycolysis and glucose oxidation genes in hearts of HFD WT and *Pgrmc1* KO mice. β-actin was used for an internal control. (**G**) Cardiac FFA level (µM) and fatty acyl-CoA levels (pmol/mg) in HFD WT and *Pgrmc1* KO mice. (**H**) Levels of glucose metabolites (unit) in HFD WT and *Pgrmc1* KO mice. (**I**) Levels of coenzyme A (CoA) and acetyl-CoA in HFD WT and *Pgrmc1* KO mice. (**J**) Levels of TCA cycle intermediates in HFD WT and *Pgrmc1* KO mice. (**K**) Ratio of ATP per ADP in HFD WT and *Pgrmc1* KO mice. Values represent means ± SD. *p < 0.05. Student’s t test was performed. Total numbers of mice used for experiment were 10 (HFD WT) and 7 (HFD *Pgrmc1* KO).

To investigate how fat-dominant conditions could affect cardiac metabolism in mitochondrial defective *Pgrmc1* KO mice, we induced HFD conditions as shown in Fig. 3A. Under these conditions, blood glucose level was decreased (p < 0.05, 67.2% vs. HFD WT), while plasma FFA levels were increased (p < 0.05, 1.33-fold vs. HFD WT) in HFD *Pgrmc1* KO mice (Fig. 3B). The expression of mtDNA was decreased (p < 0.05, 54.5% vs. HFD WT) in the hearts of HFD *Pgrmc1* KO mice (Fig. 3C). mRNA expression of TCA cycle genes, *Cs*, *Ogdh*, *Suclg2*, and *Mdh2* (p < 0.05, 28.8, 47.8, 45,7, and 64.1%, respectively, vs. HFD WT), and OXPHOS genes, *Atp5b*, *Ckmt2*, and *Ndufb5* (p < 0.05, 52.3, 60, and 69%, respectively, vs. HFD WT), were suppressed in the hearts of HFD *Pgrmc1* KO mice (Fig. 3C). mRNA expression of *Atp2a2*, *Hrc1*, and *Scn5a* was decreased (p < 0.05, 41.2, 42, and 34.7%, respectively, vs. HFD WT) in HFD *Pgrmc1* KO hearts (Fig. 3D).

Fatty acid oxidation activity was significantly lower (p < 0.05, 75.9% vs. HFD WT) in the hearts of HFD *Pgrmc1* KO mice (Fig. 3E). Expression of mRNAs of fatty acid oxidation genes, *Cpt2*, *Mcad*, and *Vlcad*, was suppressed (p < 0.05, 44.7, 53.1, and 43.1%, respectively, vs. HFD WT) in the hearts of HFD *Pgrmc1* KO mice (Fig. 3E). Protein expression of glycolysis and glucose oxidation genes, HK1, HK2, PKM2, and PDH, was increased (p < 0.05, 1.99-, 2.26-, 1.62-, and 1.65-fold, respectively, vs. HFD WT) in the hearts of HFD *Pgrmc1* KO mice (Fig. 3F).

Levels of metabolites showed a more pronounced difference in HFD *Pgrmc1* KO hearts. Levels of palmitoyl-CoA, stearoyl-CoA (C18:0), and oleoyl-CoA (C18:1) were increased (p < 0.05, 2.04-, 1.28-, and 1.6-fold, respectively, vs. HFD WT) in the hearts of HFD *Pgrmc1* KO mice (Fig. 3G). Conversely, levels of glucose, FBP, 3-PG, pyruvate, and lactate were decreased (p < 0.05, 77.7, 8.22, 35.5, 45.1, and 52.4%, respectively, vs. HFD WT) in the hearts of HFD *Pgrmc1* KO mice (Fig. 3H). Coenzyme A (CoA) was higher (1.19-fold), but the acetyl-CoA level was lower (86.2%), and the ratio of acetyl-CoA/CoA was also lower (75.9%) in the hearts of HFD *Pgrmc1* KO mice compared to those of HFD WT mice (p < 0.05, Fig. 3I). Furthermore, levels of citrate/isocitrate, fumarate, and malate were lower (p < 0.05, 10.4, 48.3, and 45.7%, respectively, vs. HFD WT) in the hearts of HFD *Pgrmc1* KO mice (Fig. 3J). As a result, the ATP/ADP ratio was suppressed (p < 0.05, 85.3%, vs. HFD WT) in the hearts of HFD *Pgrmc1* KO mice (Fig. 3K).

### *Pgrmc1* KO mice on a HFD are vulnerable to cardiac lipid accumulation

**Figure 4:**
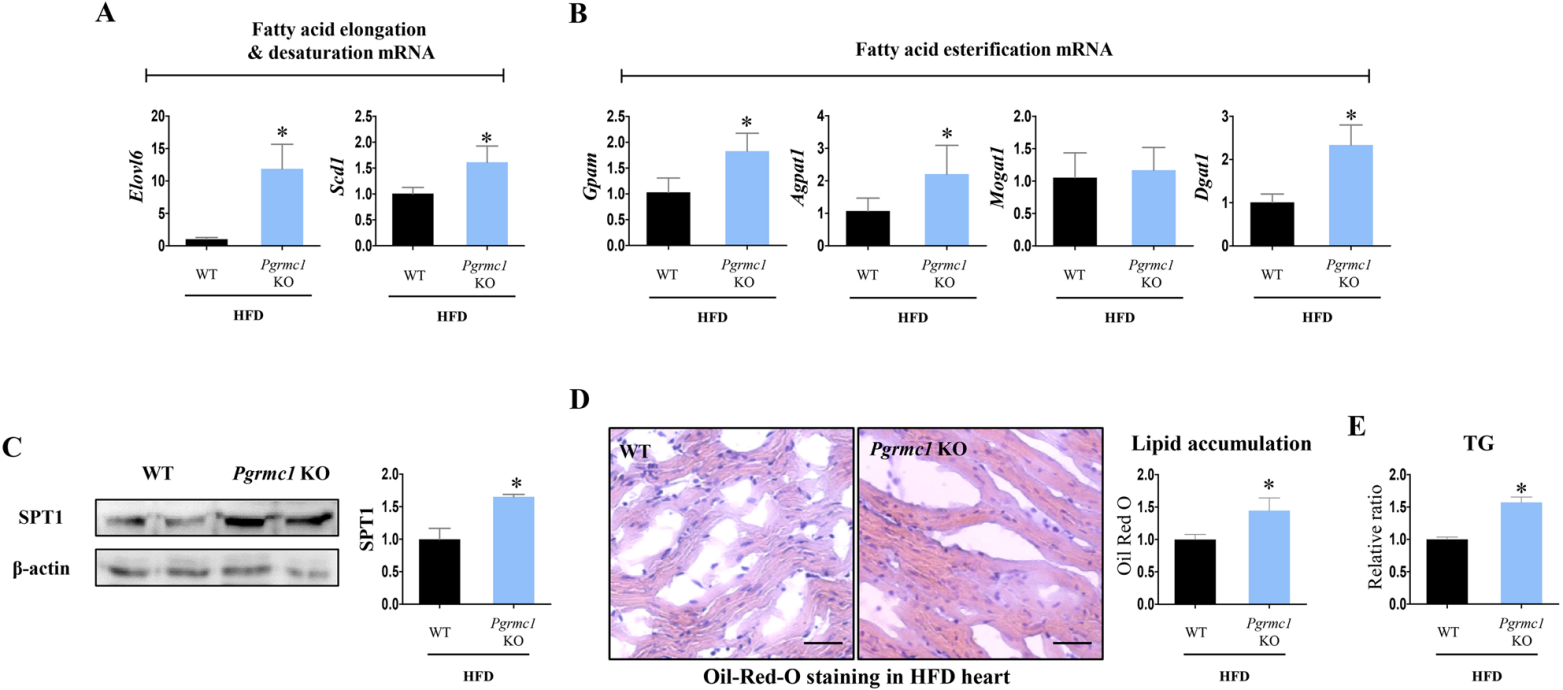
HFD *Pgrmc1* KO mice present high cardiac lipid accumulation. (**A**) mRNA expression of fatty acid elongation and desaturation genes in hearts of HFD WT and *Pgrmc1* KO mice. *Rplp0* was used for an internal control. (**B**) mRNA expression of fatty acid esterification and desaturation genes in hearts of HFD WT and *Pgrmc1* KO mice. *Rplp0* was used for an internal control. (**C**) Western blot analysis and quantification of SPT1 in hearts of HFD WT and *Pgrmc1* KO mice. β-Actin was used for an internal control. (**D**) Oil-Red-O staining and quantification in hearts of HFD WT and *Pgrmc1* KO mice (scale bar 25 µm) Positive area was measured by Image J program. Red area was set as positive. (**E**) Relative TG levels in HFD WT and *Pgrmc1* KO mice. Values represent means ± SD. *p < 0.05. Student’s t test was performed. Total numbers of mice used for experiment were 10 (HFD WT) and 7 (HFD *Pgrmc1* KO).

Excessive fatty acyl-CoA can be esterified to triacylglycerol (TG) or used for ceramide synthesis. The mRNA expression of *Elovl6*, which is responsible for fatty acid elongation, was increased (p < 0.05, 11.5-fold, vs. HFD WT) in the hearts of HFD *Pgrmc1* KO mice (Fig. 4A). The mRNA expression of *Scd1*, which is responsible for fatty acid elongation, was also increased (p < 0.05, 1.6-fold, vs. HFD WT) in the hearts of HFD *Pgrmc1* KO mice (Fig. 4A). Furthermore, the mRNA expression of fatty acid esterification genes, *Gpam*, *Agpat1*, and *Dgat1*, was increased (p < 0.05, 1.78-, 2.06-, and 2.31-fold, respectively, vs. HFD WT) in the hearts of HFD *Pgrmc1* KO mice (Fig. 4B). In addition, the protein expression of serine palmitoyltransferase 1 (SPT1), which is responsible for ceramide synthesis, was increased (p < 0.05, 1.66-fold vs. HFD WT) in the hearts of HFD *Pgrmc1* KO mice (Fig. 4C). Accordingly, Oil-Red-O staining also showed profound lipid accumulation (p < 0.05, 1.45-fold vs. HFD WT) in the hearts of HFD *Pgrmc1* KO mice (Fig. 4D). TG level was also higher (p < 0.05, 1.57-fold vs. HFD WT) in HFD *Pgrmc1* KO hearts (Fig. 4E).

### *Pgrmc1* KO mice on a HFD present various cardiac lipotoxic markers

**Figure 5:**
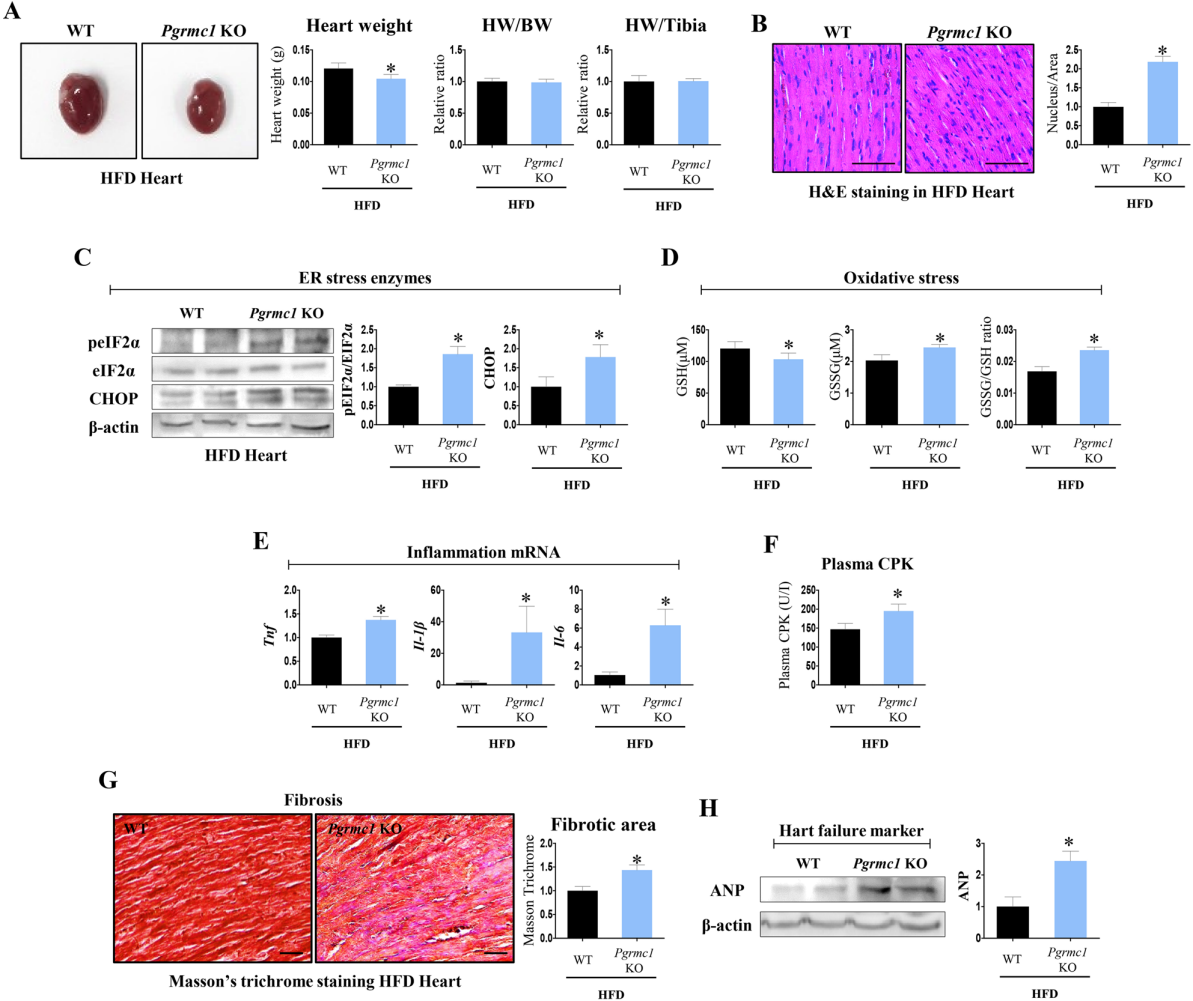
*Pgrmc1* KO mice shows lipotoxicity in HFD condition. (**A**) Gross image of hearts of HFD WT and *Pgrmc1* KO mice. Heart weight (HW), HW per body weight (BW) and HW per tibia length were measured. (**B**) H&E staining of hearts of HFD WT and *Pgrmc1* KO mice (scale bar 50 µm). Nucleus per area was measured by Image J program. (**C**) Western blot analysis and quantification of ER stress genes in hearts of HFD WT and *Pgrmc1* KO mice. β-Actin was used for an internal control. (**D**) Cardiac levels of reduced (GSH) and oxidized (GSSG) glutathione (µM), and ratio of GSSG:GSH in HFD WT and *Pgrmc1* KO mice. (**E**) mRNA expression of pro-inflammatory genes in hearts of HFD WT and *Pgrmc1* KO mice. *Rplp0* was used for an internal control. (**F**) Plasma CPK (U/I) level of HFD WT and *Pgrmc1* KO mice. (**G**) Masson trichrome staining of hearts of HFD WT and *Pgrmc1* KO mice (scale bar 30 µm). Positive area was measured by Image J program. Blue fibroblasts were set as positive. (**H**) Western blot analysis and quantification of ANP in hearts of HFD WT and *Pgrmc1* KO mice. β-Actin was used for an internal control. Values represent means ± SD. *p < 0.05. Student’s t test was performed. Total numbers of mice used for experiment were 10 (HFD WT) and 7 (HFD *Pgrmc1* KO).

While the cardiac lipid accumulation and low ATP production were observed in HFD *Pgrmc1* KO mice, the heart did not show hypertrophy, as shown in Fig. 5A. Conversely, the heart weight (HW) was decreased (p < 0.05, 86.5% vs. HFD WT) in HFD *Pgrmc1* KO mice.

However, the ratio of HW/body weight (BW) was not decreased in HFD *Pgrmc1* KO mice. Consistently, HW/tibia length was similar between HFD WT and *Pgrmc1* KO mice (Fig. 5A). In H&E staining, number of nuclei per area was increased (p < 0.05, 2.18-fold vs. HFD WT) in the hearts of HFD *Pgrmc1* KO mice (Fig. 5B), suggesting the cardiac cell size is smaller.

As indicators of ER stress, the ratio of pEIF2α/EIF2α protein and the expression of CHOP protein were increased (p < 0.05, 1.86- and 1.78-fold, respectively, vs. HFD WT) in the hearts of HFD *Pgrmc1* KO mice (Fig. 5C). Furthermore, oxidative stress was increased in the hearts of HFD *Pgrmc1* KO mice; the level of reduced glutathione (GSH) was decreased (p < 0.05, 85.9% vs. HFD WT), while the levels of oxidized glutathione (GSSG) and the GSSG/GSH ratio had increased (p < 0.05, 1.2- and 1.4-fold, respectively, vs. HFD WT), as shown in Fig. 5D. The mRNA expression of pro-inflammatory cytokine genes, including *Tnf*, *Il-1β*, and *Il-6,* was increased (p < 0.05, 1.37-, 25-, and 6.02-fold, respectively, vs. HFD WT) in the hearts of HFD *Pgrmc1* KO mice (Fig. 5E). The level of plasma creatine phosphokinase (CPK) was increased (p < 0.05, 1.33-fold vs. HFD WT) in HFD *Pgrmc1* KO mice (Fig. 5F). Abnormal stress led to the induction of cardiac fibrosis, as observed by Masson’s trichrome staining. A significant increase (p < 0.05, 1.44-fold vs. HFD WT) in fibroblasts (blue staining) was observed in the hearts of HFD *Pgrmc1* KO mice (Fig. 5G). Furthermore, as a cardiac failure marker, the expression of ANP was increased (p < 0.05, 2.44-fold vs. HFD WT) in the hearts of HFD *Pgrmc1* KO mice (Fig. 5H). Based on these results, we concluded that the metabolic phenotype of *Pgrmc1* KO mice leads to cardiac lipotoxicity under HFD conditions, but does not trigger cardiac hypertrophy.

### Pgrmc1 increases mitochondrial respiration and fatty acid oxidation in H9c2 cells

**Figure 6:**
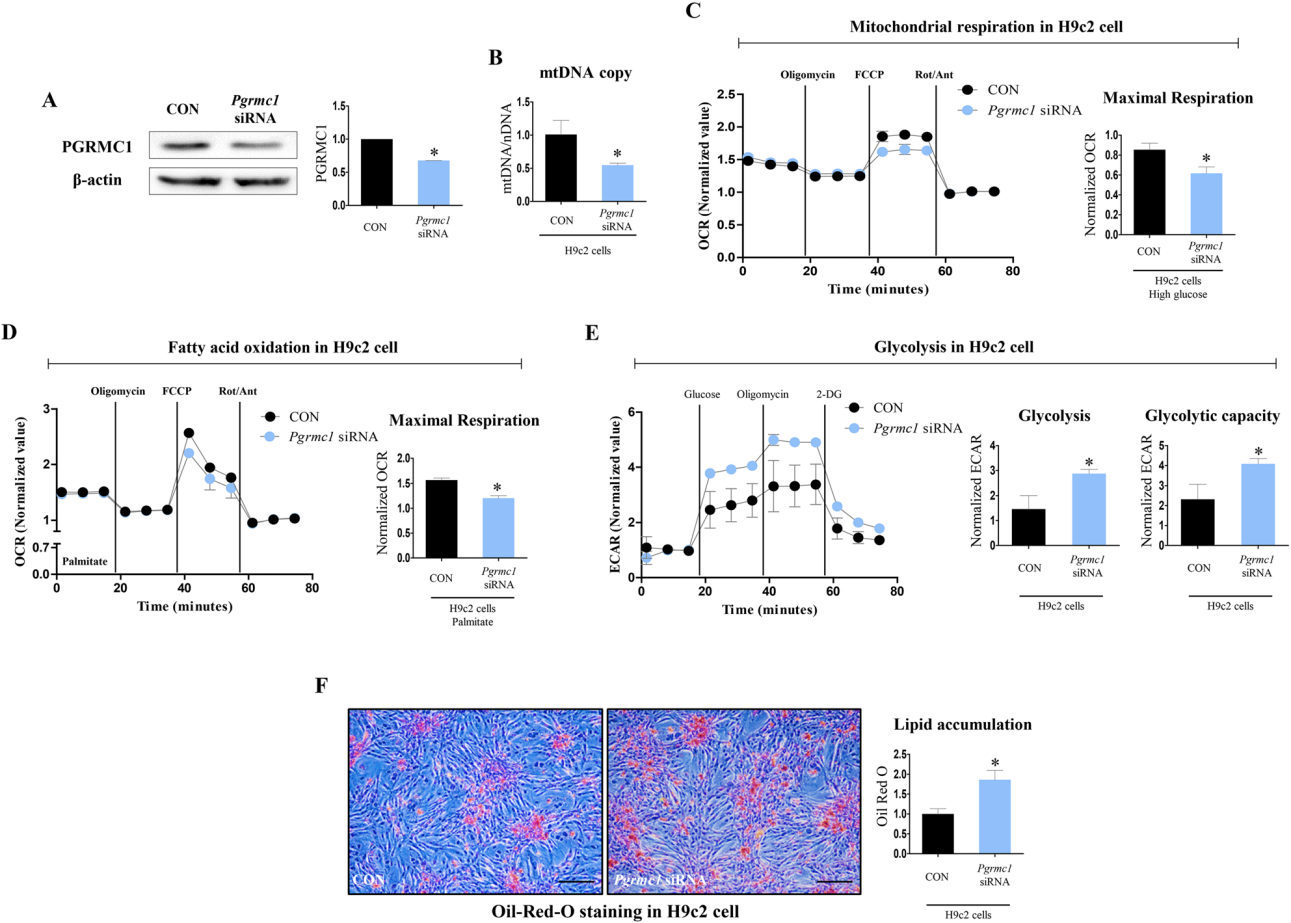
*Pgrmc1* increases mitochondrial respiration and fatty acid oxidation in H9c2 cells. (**A**) Western blot analysis and quantification of PGRMC1 in H9c2 cells. β-Actin was used for an internal control. (**B**) Copies of mitochondrial DNA (mtDNA) in H9c2 cells. Nuclear DNA (nDNA) was used for an internal control. (**C**) Mitochondrial respiration measured by using flux analyzer in H9c2 cells. Values were normalized to baseline. (**D**) Glycolysis rate measured by using flux analyzer in H9c2 cells. Values were normalized to baseline. (**E**) Fatty acid oxidation rate measured by mitochondrial stress test using flux analyzer in palmitate-BSA treated condition in H9c2 cells. Values were normalized to baseline. (**F**) Oil-Red-O staining in palmitate-BSA treated condition in H9c2 cells (scale bar 100 µm). Positive area was measured by Image J program. Red area was set as positive. Values represent means ± SD. *p < 0.05. Student’s t test was performed. All experiments were repeated at least 3.

H9c2 cells were transfected with *Pgrmc1* siRNA, and the protein expression of PGRMC1 was suppressed (p < 0.05, 67.8% vs. *control* siRNA (CON)) in the *Pgrmc1* siRNA group (Fig. 6A). The number of mtDNA copies was decreased (p < 0.05, 54.1% vs. CON) in the *Pgrmc1* siRNA group (Fig. 6B). Using the seahorse flux analyzer in H9c2 cells, we monitored the oxygen consumption rate (OCR), wherein other endocrine factors were not considered.

The level of maximal respiration was suppressed (p < 0.05, 72.2% vs. CON) in the *Pgrmc1* siRNA group (Fig. 6C). To measure the rate of fatty acid oxidation, palmitate was conjugated with CD-BSA (charcoal dextran-bovine serum albumin) and used for treatment. The level of maximal respiration was decreased (p < 0.05, 76.8% vs. CON) in the *Pgrmc1* siRNA group (Fig. 6D). Conversely, the levels of glycolysis and glycolytic capacity, which were measured by the extracellular acidification rate (ECAR), were increased (p < 0.05, 1.97- and 1.76-fold, respectively, vs. CON) in the *Pgrmc1* siRNA group (Fig. 6E). In the palmitate-BSA-treated condition, the positive area for Oil-Red-O staining was increased (p < 0.05, 1.86-fold vs. CON) in the *Pgrmc1* siRNA group (Fig. 6F). According to the in vitro results, we confirmed that *Pgrmc1* is involved in mitochondrial metabolism, especially for fatty acid oxidation in cardiac cells.

## Discussion

Obesity is characterized by the gain of body weight and fat mass. When fat mass accumulates around abdominal organs, visceral obesity occurs, leading to susceptibility to cardiovascular conditions^14^. Regarding physiological aspects of the heart, cardiac TG accumulation is a common characteristic of most animal models of obesity, as the heart promotes fatty acid uptake even in a lipotoxic state^15^. Therefore, fatty acid disposal in the heart is crucial to maintain intracardiac lipid balance, but fatty acid oxidation is limited under condition of failing heart. Compensatory glucose utilization is activated, but ATP production cannot be fully replenished. Consequently, the lack of fatty acid oxidation leads to impairment of cardiac contractility^16^. In a HFD mouse model, *Pgrmc1* KO hearts presented lipid accumulation and suppressed ATP production. This was due to impaired fatty acid oxidation and mitochondrial dysfunction of Pgrmc1 KO hearts.

Cardiac mitochondrial function is crucial for energy metabolism because the heart relies on oxidative phosphorylation for energy production^17^. Cardiac cells possess large numbers of mitochondria^18^, and ATP produced by mitochondrial oxidative phosphorylation is used for cardiac contractile function, as it is hydrolyzed to ADP^19^. *Pgrmc1* KO hearts in resting, refed, and HFD conditions all presented low expression of mtDNA and mRNA expression of contractile markers. Furthermore, mRNA expression of TCA cycle genes and OXPHOS genes was all decreased in *Pgrmc1* KO hearts. Our results show that *Pgrmc1* KO hearts are phenotypically lacking mitochondrial function.

In a pathological murine model, a high-fat, high-sucrose (HFHS) diet causes cardiac mitochondrial dysfunction and decreased cardiac ATP production^20^. From a biochemical perspective, ATP is generated in the electron transport system by NADH and FADH_2_, which are produced in the TCA cycle. Fatty acid and glucose oxidation both produce acetyl-CoA, which drives the TCA cycle, and the acetyl-CoA/CoA ratio indicates acetyl-CoA production^21^. Fatty acid oxidation is the primary pathway for ATP production in the heart, while glucose oxidation is the more efficient pathway^19^. Fatty acid oxidation inhibits glucose oxidation, thereby balancing cardiac metabolism^19,22^. While *Pgrmc1* KO hearts lack fatty acid oxidation activity, the acetyl-CoA production was maintained in a resting and refed state. This result can be interpreted in two ways; (1) decrease of fatty acid oxidation was not enough to trigger a physiological response. (2) acetyl-CoA from glucose source compensated the level. Especially in the refed state, where glucose is enriched, the increased expression of glycolysis and glucose oxidation and low level of glucose metabolites in *Pgrmc1* KO hearts were observed, suggesting compensatory recovery of acetyl-CoA from glucose. Conversely, in fat-rich condition (HFD), *Pgrmc1* KO hearts presented compensatory response from glucose according to the induction of glucose metabolic pathway and low glucose metabolite levels, but acetyl-CoA production was suppressed. Furthermore, TCA cycle intermediates levels were low, especially for citrate/isocitrate, fumarate, and malate. According to the low capacity of ATP production in HFD *Pgrmc1* KO hearts, these comparative results suggest that lack of fatty acid oxidation activity in fat-enriched conditions leads Pgrmc1 KO hearts to pronounced impairment of ATP production. While Pgrmc1 KO hearts could spend the cellular fatty acid in resting and refed conditions, they failed to fully use the cellular fatty acid in long-term HFD conditions.

Low fatty acid usage can result in excessive residual fatty acyl-CoA and induce lipid accumulation in the heart. Palmitoyl-CoA is the first form of fatty acyl-CoA synthesized from acetyl-CoA, which is converted to stearoyl-CoA by elongation. Scd1 desaturates both palmitoyl-CoA and stearoyl-CoA to form oleoyl-CoA. Afterward, desaturated fatty acyl-CoA is esterified through catalysis by a series of enzymes, including Gpam, Agpat1, Mogat1, and Dgat1, to form triglycerides^8^. Meanwhile, ceramide also would be synthesized in the condition of lipid overload by conjugation of serine and palmitoyl CoA. The first and rate-limiting step of ceramide synthesis is controlled by SPT1^23^. When the heart of HFD *Pgrmc1* KO mice has high levels of fatty acyl-CoA due to limited fatty acid oxidation activity, they could be successfully esterified to triglycerides or used for ceramide synthesis considering the expression of genes. Accordingly, lipid accumulation was observed in the heart of HFD *Pgrmc1* KO mice.

Lipotoxicity triggered by lipid is controversial as it differs by lipid species, including TG, DAG, and ceramide^24^. Nonetheless, as the heart is not a conventional lipid-storing organ, lipid overload in the heart is still unusual and can cause cardiac abnormalities^25^. Lipid-induced heart failure accompanies hypertrophy, fibrosis, inflammation, and mitochondrial dysfunction, which lead to contractile dysfunction^26^. HFD *Pgrmc1* KO hearts was not linked with cardiac hypertrophy according to similar HW/BW and HW/tibial length to those of HFD WT mice. Instead, HW and BW of HFD *Pgrmc1* KO mice were both low. As weight gain during the HFD period was similar between WT and *Pgrmc1* KO mice (Fig. S1), *Pgrmc1* KO mice are phenotypically lighter than WT mice. Conversely, HFD *Pgrmc1* KO hearts induced various heart failure markers. Increased pEIF2α and CHOP expression, high GSSG and low GSH levels, and increased inflammatory gene expression in *Pgrmc1* KO heart are indicative of heart failur^27–30^. Plasma CPK was also increased, which serves as a cardiac damage marker^31^. Finally, fibrosis^27^ and cardiac failure markers^32^ suggest the cardiac failure of *Pgrmc1* KO mice.

Our hypothesis that was based on the in vivo study was confirmed in an in vitro study. *Pgrmc1* knockdown resulted in low mtDNA copy number and suppression of mitochondrial respiration in H9c2 cells. Furthermore, consistent to in vivo study, induction of glycolysis was observed in the *Pgrmc1* knockdown group of H9c2 cells. Finally, suppression of fatty acid oxidation resulted in remarkable lipid accumulation in *Pgrmc1* knockdown H9c2 cells.

Collectively, this study highlights the role of Pgrmc1 as a metabolic modulator of cardiac health. Since suppression of fatty acid oxidation without decreasing mitochondrial biogenesis is observed in the early stages of heart failure^33^, and characteristic mitochondrial dysfunction is seen in the late stages of heart failure^34^, individuals with low cardiac *Pgrmc1* expression might be prone to mitochondrial impairment and progression to heart failure. According to our study, induction of *Pgrmc1* expression improves the fundamental cardiac energy capacity and reduces cardiac lipid accumulation by increasing fatty acid oxidation and mitochondrial respiration. This is important because a fundamental increase in ATP production capacity is suggested for the targeted treatment of cardiomyopathy^35^.

## Methods

### Animals

C57BL/6J mice were housed in a pathogen-free facility at Chungnam National University under a standard 12 h light:12 h dark cycle and fed standard chow or high-fat diet, with water provided ad libitum. All animal experiments were carried out in accordance with the National Institutes of Health Guide for the Care and Use of Laboratory Animals (Republic of Korea), and in compliance with ARRIVE guidelines. Animal experiment was approved by the Institutional Animal Care and Use Committee of Chungnam National University (IACUC; approval no. CNU-01145). As reported previously^9^, *Pgrmc1* KO mice and wild-type littermates were used in this study. Refed conditions were induced by 6 h of feeding after 18 h of fasting. HFD conditions were induced by injecting streptozotocin (30 mg/kg) and feeding a high-fat diet for 8 weeks according to previous studies^36^. The high-fat diet (#D12492, Research Diets, Inc., New Brunswick, NJ) was composed of carbohydrate (20% kcal), protein (20% kcal), and fat (60% kcal).

### Fatty acid oxidation activity

Fatty acid oxidation kit (E-141) was obtained from Biomedical Research (BMR, Buffalo, USA). All tissue was processed according to the manufacturer’s protocol. Values were normalized by protein concentration.

### Measurements of biochemical values

All plasma samples were diluted 1/5-fold with PBS. Plasma FFA levels were measured employing a commercial kit (BM-FFA100) obtained from Biomax (Seoul). Blood glucose levels were measured by tail snipping using an Accu-Chek Active (Roche, 07124112) kit.

### Measurements of cellular mitochondrial respiration, glycolysis and fatty acid oxidation

H9C2 cells were transfected and then transferred to a seahorse cell culture plate. The cells were then cultured in DMEM serum-starved medium [i.e., without the addition of fetal bovine serum (FBS)] for 5–6 h to remove endogenous hormones. For only glycolysis stress test, cells were further incubated in DMEM low-glucose medium [glucose 50 mg/dL without FBS]. Before the experiment, the cells were decarboxylated for 40 min to 1 h in XFp medium (103575-100, Agilent Technologies) containing the same amount of glutamine, sodium pyruvate, and glucose as that of the medium in which cells were grown. For the mitochondrial stress test (mitochondrial respiration and fatty acid oxidation), oligomycin (2 µM), FCCP (5 µM), and rotenone/antimycin (0.5 µM) were used, and oxygen consumption rate (OCR) was measured. For fatty acid oxidation measurement, BSA(34 µM)-conjugated palmitate (200 µM) was added to seahorse cell culture plate. For the glycolysis stress test, glucose (25 mM), oligomycin (2 µM), and 2-deoxy-d-glucose (2-DG, 100 µM) were used, and extracellular acidification rate (ECAR) was measured. Seahorse XFp analyzer (Agilent Technologies), Seahorse XFp, XFp FluxPak (103022-100, Agilent Technologies), and XFp Cell Mito Stress Test kit (103010-100, Agilent Technologies) were used for analysis.

### Measurements of metabolites (HPLC–MS/MS)

Standard metabolites and internal standards were purchased from Sigma-Aldrich and CDN isotopes. All solvents including water were purchased from J. T. Baker. Tissue was homogenized using TissueLyzer (Qiagen) after adding 400 μL chloroform/methanol (2/1, v/v) and 100 μL internal standard solution containing 10 μM 13C5-glutamine for metabolites related to energy metabolism and 5 μM malonyl-13C3 CoA for fatty acyl CoAs. Metabolites were extracted from aqueous phase by liquid–liquid extraction^37^. The aqueous phase was dried using vacuum centrifuge, and the sample was reconstituted with 50 μL of H_2_O/acetonitrile (50/50 v/v) prior to LC–MS/MS analysis. Metabolites were analyzed with LC–MS/MS equipped with 1290 HPLC (Agilent), Qtrap 5500 (ABSciex), and reverse phase columns (Synergi fusion RP 50 × 2 mm for metabolites related to energy metabolism and Zorbax Extend-C18 3.5 μm, 2.1 × 12.5 mm for fatty acyl-CoA). Multiple reaction monitoring (MRM) was used, and the extracted ion chromatogram (EIC) corresponding to the specific transition for each metabolite was used for quantitation. Area under the curve of each EIC was normalized to that of EIC of internal standard, and used for quantitation.

### Measurement of cardiac TG

Lipid extraction from heart was done with Folch method. Briefly, tissue was homogenized with beads and 0.9% NaCl solution. Homogenates were mixed with chloroform and methanol and incubated. Chloroform and distilled water were added and homogenates were centrifuged, and lower phase was collected. Steps after homogenization were 3 times repeated. Samples were dried and dissolved in chloroform and 2-propanol. TG level was analyzed by TG measurement solution (AM157S-K, Asan-Set).

### Measurement of cardiac FFA

Commercial kit (BM-FFA100) for FFA measurement was obtained from Biomax. Samples were processed according to manufacturer’s protocol, and the level of cardiac FFA was measured.

### RNA isolation, reverse transcription, and qRT-PCR

Heart was homogenized with TRIzol Reagent (Thermo Fisher Scientific, MA, USA) and the homogenate was mixed with chloroform (C2432, Sigma). After centrifugation, supernatant was mixed with isopropanol (1.09634.1011, Merck) and incubated for overnight on −20 °C. RNA pellet was attained by centrifugation, and the pellet was washed once with 70% ethanol and dissolved in DEPC (E174, Amresco)-treated water. cDNA was acquired by using a Reverse Transcriptase Kit (SG-cDNAS100, Smartgene, United Kingdom). mRNA expression was evaluated by real-time PCR using cDNA and specific primers (shown in Table 1). Excel Taq Q-PCR Master Mix (SG-SYBR-2000, Smartgene) and Stratagene Mx3000P (Agilent Technologies) were used to perform real-time PCR.

**Table 1:** Primers used for real-time PCR.

Gene name	Upper primer (5′–3′)	Lower primer (5′–3′)	Species
*Cpt2*	CAG CAC AGC ATC GTA CCC A	TCC CAA TGC CGT TCT CAA AAT	Mouse
*Mcad*	AGG TTT CAA GAT CGC AAT GG	CTC CTT GGT GCT CCA CTA GC	Mouse
*Vlcad*	TAT CTC TGC CCA GCG ACT TT	TGG GTA TGG GAA CAC CTG AT	Mouse
*Cs*	CCT GAG TGC CAG AAA ATG CTG	CCA CAT GAG AAG GCA GAG CT	Mouse
*Aco2*	ACA AGT GGG ACG GCA AAG AC	AGC ATT GCG TAC AGA GTT GGC	Mouse
*Idh3a*	TGC TTC GCC ACA TGG GAC TT	CGT TGC CTC CCA GAT CTT TT	Mouse
*Ogdh*	AAT GCT GAG CTG GCC TGG TG	TCA GGT GTG TTT TCT TGT TGC C	Mouse
*Suclg2*	CTG TGC CAT CAT TGC CAA CG	ATG GGG AGT CCG CTG CTC TT	Mouse
*Sdhd*	TCA GAC CCG CTT ATG TGT CA	CAG CCC CAA GAG CAG AAC AC	Mouse
*Fh1*	GTG GAA GTT CAC AAG GTC CTG	GGA CTT GCT GAA CGT AAC CAC	Mouse
*Mdh2*	ATG CTG GAG CCC GCT TTG TC	CAG GGA TAG CCT CGG CAA TC	Mouse
*Atp5b*	GTA CTG GAT TCA GGG GCA CC	CTA TGA ACT CAG GAG CCT CAG C	Mouse
*Ckmt2*	GTG CGG ACT ACC CTG ACC TTC	CCG TAG GAT GCT TCA TCA CCC	Mouse
*Ndufb5*	CGA GCT TGC AGA AAT CCC AGA AGG C	GTC CAT CAC CTC GGG CAC GCA TCA G	Mouse
*Slc25a4*	CGG CTC CTT GCA GGC TGT GTG	CAA TGA TGC CTT TGT ACT GC	Mouse
*Atp2a2*	GAG AAC GCT CAC ACA AAG ACC	CAA TTC GTT GGA GCC CCA T	Mouse
*Hrc1*	CAA CCG ATG TAC CGA ATG TGA	GGT AGC AGA ATT GAC AGT GCT G	Mouse
*Scn5a*	TGC TGA ATA AGG GCA AAA CCA	GCT GAA GAG CGA ATG TAC CAA AA	Mouse
*Elovl6*	AGC AAA GCA CCC GAA CTA GG	CCA GGA GTA CAG GAG CAC A	Mouse
*Scd1*	CTG TTC GTT AGC ACC TTC TTG	CAG AGT AGT CGA AGG GGA AG	Mouse
*Gpam*	AGA GGC TTC TAG GTC CCC TG	TTC ACG AGA CAG TAT GTG GC	Mouse
*Agpat1*	TAA GAT GGC CTT CTA CAA CGG C	CCA TAC AGG TAT TTG ACG TGG AG	Mouse
*Mogat1*	TGG TGC CAG TTT GGT TCC AG	TGC TCT GAG GTC GGG TTC A	Mouse
*Dgat1*	TCC GTC CAG GGT GGT AGT G	TGA ACA AAG AAT CTT GCA GAC GA	Mouse
*Tnf*	CCT GTA GCC CAC GTC GTA G	GGG AGT AGA CAA GGT ACA ACC C	Mouse
*Il-1β*	GAA ATG CCA CCT TTT GAC AGT G	CTG GAT GCT CTC ATC AGG ACA	Mouse
*Il-6*	CTG CAA GAG ACT TCC ATC CAG	AGT GGT ATA GAC AGG TCT GTT GG	Mouse
*mtDNA*	CCT ATC ACC CTT GCC ATC AT	GAG GCT GTT GCT TGT GTG AC	Mouse
*nDNA*	ATG GAA AGC CTG CCA TCA TG	TCC TTG TTG TTC AGC ATC AC	Mouse
*mtDNA*	AAG TGG CTG TGC AGA CAT TC	TCT GTC TTT GAT TCC TGC CT	Rat
*nDNA*	TCT CCT ACT TGG ATA ACT GTG G	GGC GAC TAC CAT CGA AAG TTG	Rat

### Western blotting

Protein samples were quantified by Bradford assay using PRO-Measure solution (Intron, #21011) and subjected to protein electrophoresis (SDS-PAGE). Gels were blotted by wet transfer using a Bio-Rad Power Pac at 350 V for 1 or 2 h. Membranes were blocked and incubated with primary antibodies for overnight at 4 ℃. After washing, the membranes were incubated with secondary antibodies overnight at 4 °C. Next, after a washing step, the protein bands were detected using an ECL kit (XLS025-0000, Cyanagen) on the ChemiDoc system (Fusion Solo, Vilber Lourmat).

The primary antibodies used were: β-actin [Rabbit polyclonal antibody (Rab Poly Ab), Santa Cruz, sc-130656], HK1, HK2, PKM2, PDH [Rab Poly Ab, Cell Signaling Technology (CST), #8337], eIF2α (Rab Poly Ab, CST, #9722), peIF2α [Rabbit monoclonal antibody (Rab mono Ab), CST, #3597], CHOP (Mouse Monoclonal antibody, Invitrogen, #MA1-250), ANP (Rab poly Ab, Abcam, ab14348), SPT1 (Rab poly Ab, ABclonal, A6750), and PGRMC1 (Rab mono Ab, CST, #13856). The secondary antibodies used were: Mouse anti-rabbit IgG (211-032-171, Jackson ImmunoResearch, 1:5000) and Goat anti-mouse IgG antibody (BS-0296G-HRP, Bioss, 1:5000).

### Masson’s trichrome staining

Sections were cut from paraffin blocks and processed using a commercial kit (MST-100 T, BIOGNOST). The fibrotic area was quantified by analysis with Image J program.

### Cell culture

All cell culture reagents were purchased from Welgene (Gyeongsan, Korea). H9c2 rat cardiomyocte cells were maintained at 37 °C in a 5% CO_2_ atmosphere in DMEM (Welgene, LM001-05) supplemented with 5% (v/v) FBS, penicillin (100 U/mol), and streptomycin (100 μg/mL). All the cell experiments were repeated at least 3 times.

### PGRMC1 knockdown in vitro

For *PGRMC1* knockdown, siRNA transfection was performed using the Lipofectamine 2000 reagent (11668-027, Thermo Fisher) according to the manufacturer’s protocol. Negative control siRNA and *PGRMC1* siRNA #1 and #2 were purchased from Bioneer (Daejeon, Korea). The sense sequences of *PGRMC1* siRNA #1 and #2 were 5′-CAGUACAGUCGCUAGUCAA-3′ and 5′-CAGUUCACUUUCAAGUAUCA-U-3'.

### Statistical analysis

Data are reported as mean ± SD. Student’s t test was performed using Graph Pad Software (GraphPad Inc., San Diego, CA, USA).

## Supplementary Information

Supplementary Information 1.
