# Supplementary material for: Progesterone receptor membrane component 1 reduces cardiac steatosis and lipotoxicity via activation of fatty acid oxidation and mitochondrial respiration
Source: Sci Rep. 2021 Apr 22;11:8781. doi: 10.1038/s41598-021-88251-2 (PMC8062525; doi:10.1038/s41598-021-88251-2)
Supplement: Supplementary file 1 — Supplementary Information 1. [file 41598_2021_88251_MOESM1_ESM.pdf]

Supplemental material

Progesterone receptor membrane component 1 reduces cardiac steatosis and lipotoxicity *via* activation of fatty acid oxidation and mitochondrial respiration

Sang R. Lee[1], Jun H. Heo[1], Seong Lae Jo[1], Globinna Kim[2], Su Jung Kim[2], Hyun Ju Yoo[2], Kyu-Pil Lee[1], Hyo-Jung Kwun[1], Hyun-Jin Shin[1], In-Jeoung Baek[2*], and Eui-Ju Hong[1*]

[1]College of Veterinary Medicine, Chungnam National University, Daejeon 34134, Republic of Korea; [2]Department of Convergence Medicine, University of Ulsan College of Medicine, Asan Medical Center, Seoul 05505, Republic of Korea

Fig.S1

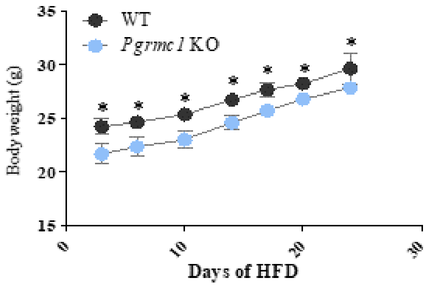

Figure S1. Body weight monitoring during HFD feeding. Values represent means ± SD. *, $p<0.05$. Student's t-test was performed. Total numbers of mice used for experiment were 5 (HFD WT) and 5 (HFD *Pgrmc1* KO).

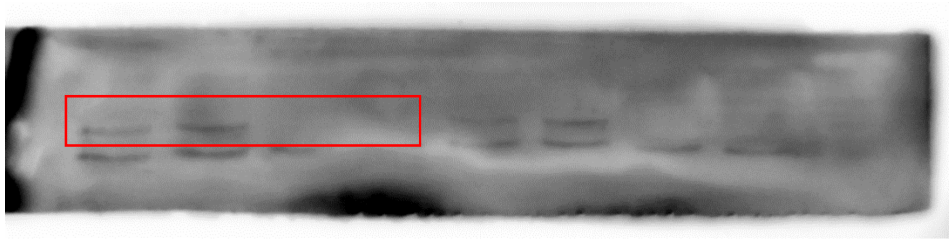
Fig. 1F
PGRMC1

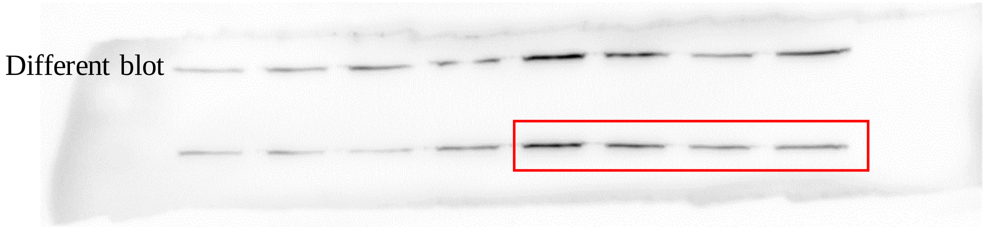

Fig. 1F
HK1

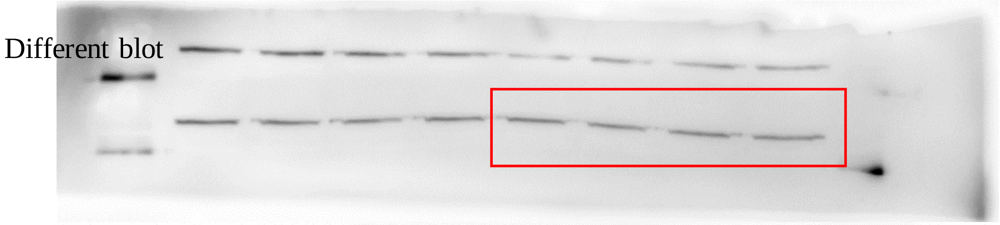

Fig. 1F
HK2

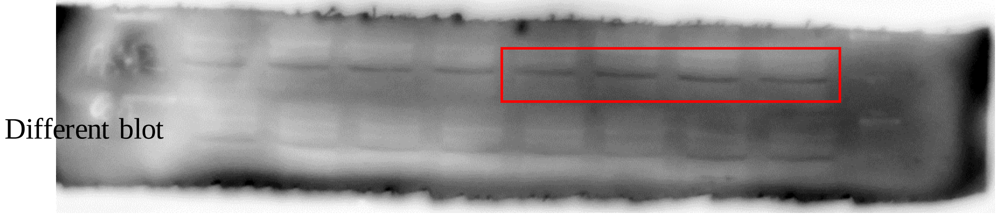

Fig. 1F
PKM2

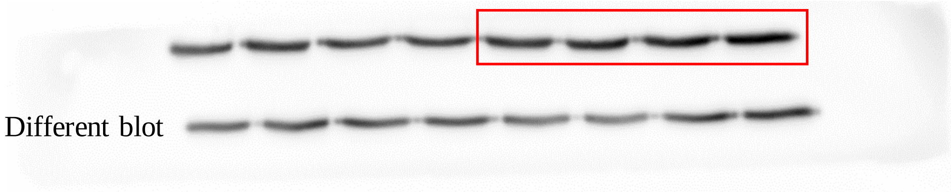

Fig. 1F
PDH

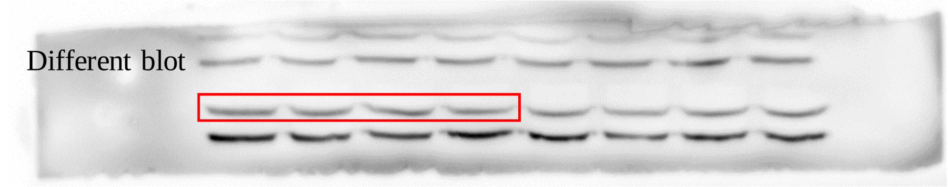

Fig. 1F
β-actin

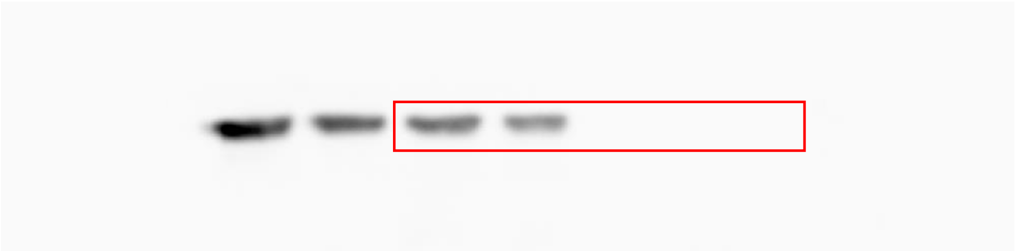

Fig. 2F
PGRMC1

Different blot

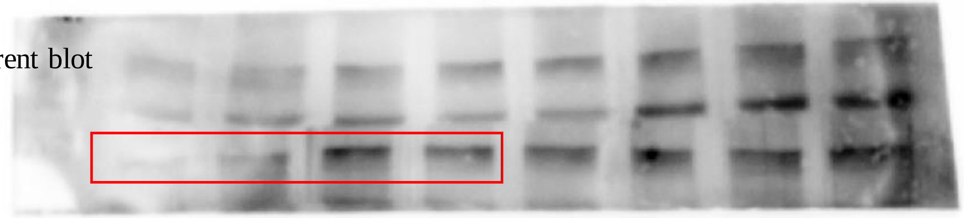

Fig. 2F
HK1

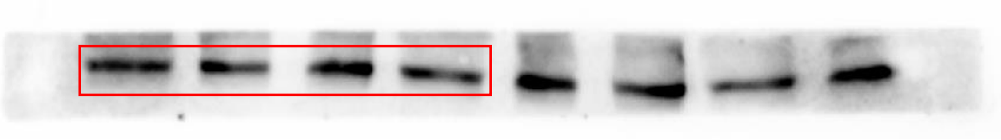

Fig. 2F
HK2

Different blot

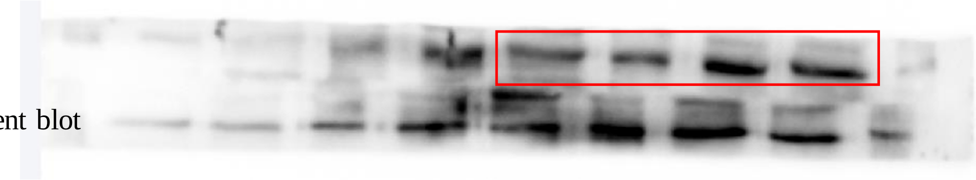

Fig. 2F
PKM2

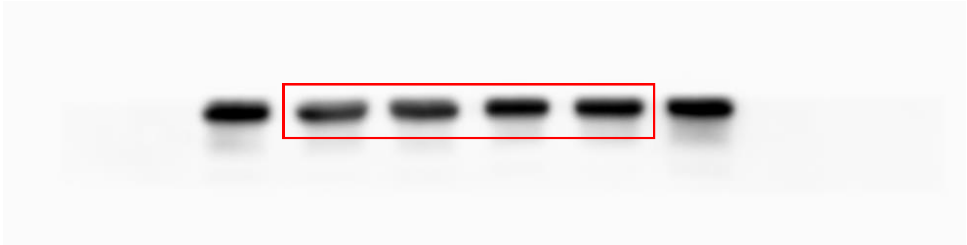

Fig. 2F
PDH

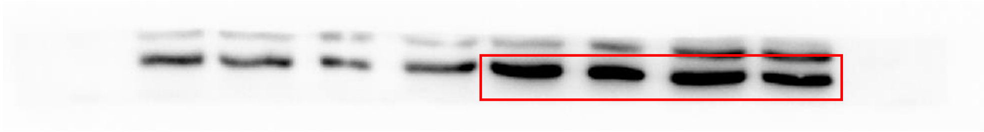

Fig. 2F
β-actin

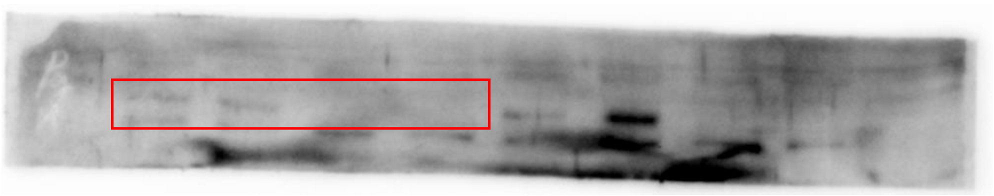

Fig. 3F
PGRMC1

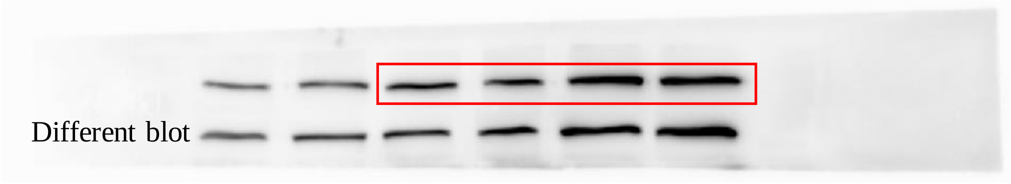

Fig. 3F
HK1

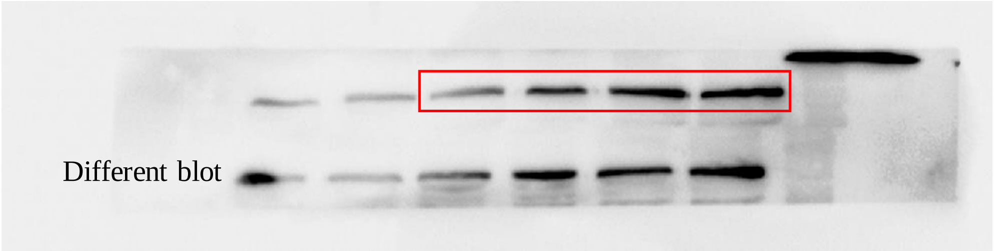

Fig. 3F
HK2

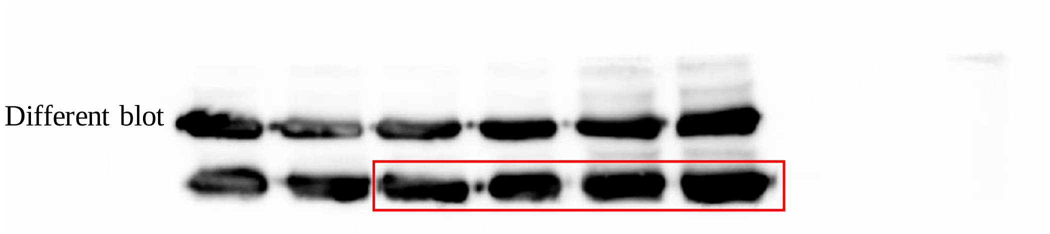

Fig. 3F
PKM2

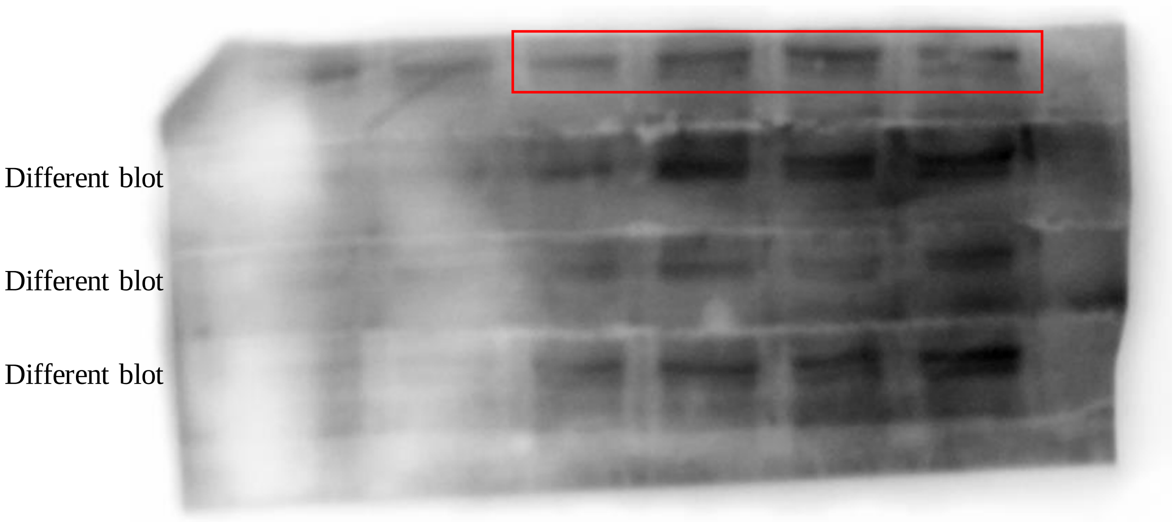

Fig. 3F
PDH

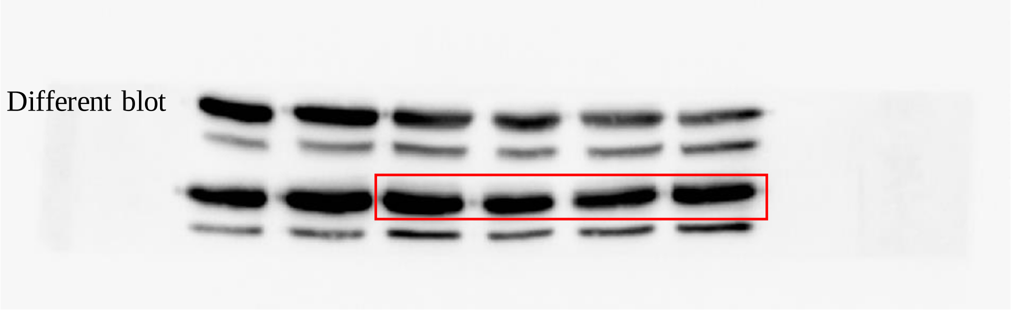

Fig. 3F
β-actin

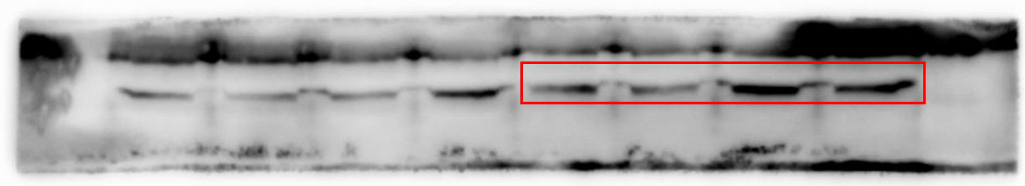

Fig. 4C
SPT1

Different blot

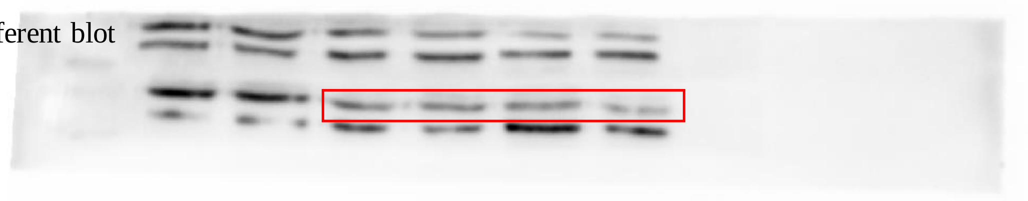

Fig. 4C
β-actin

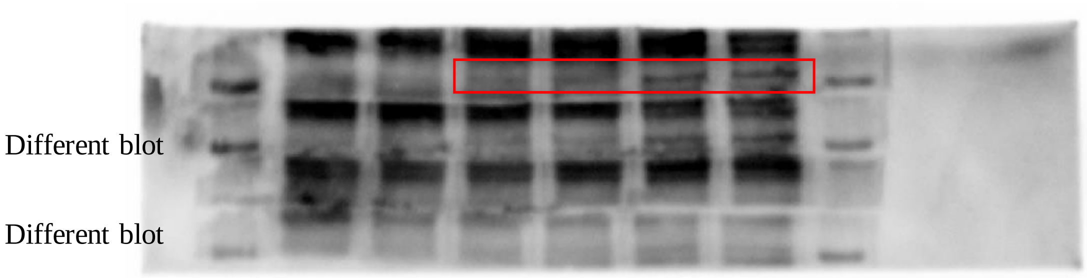

Fig. 5C
peIF2α

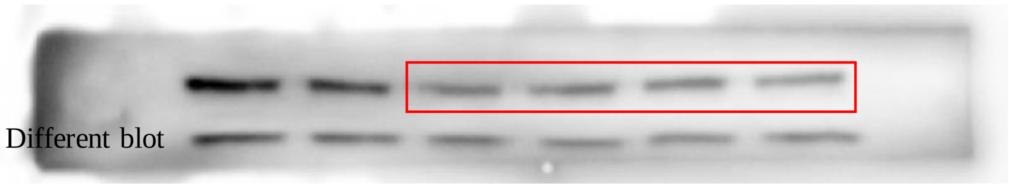

Fig. 5C
eIF2α

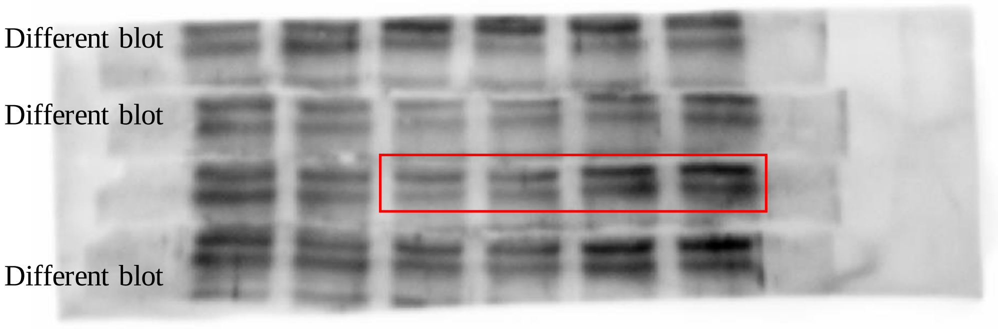

Fig. 5C
CHOP

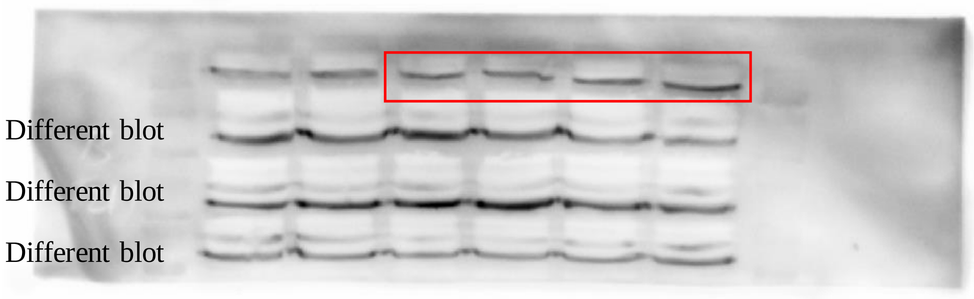

Fig. 5C
β-actin

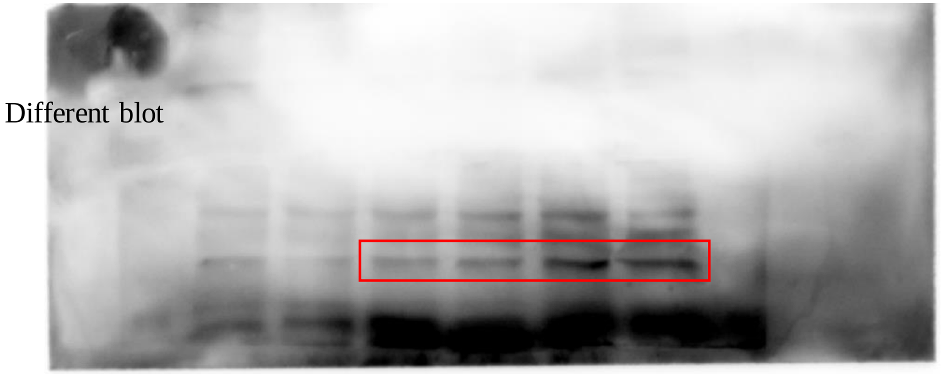

Fig. 5H
ANP

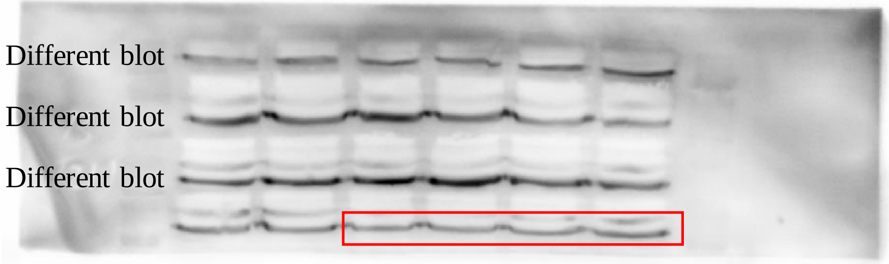

Fig. 5H
β-actin

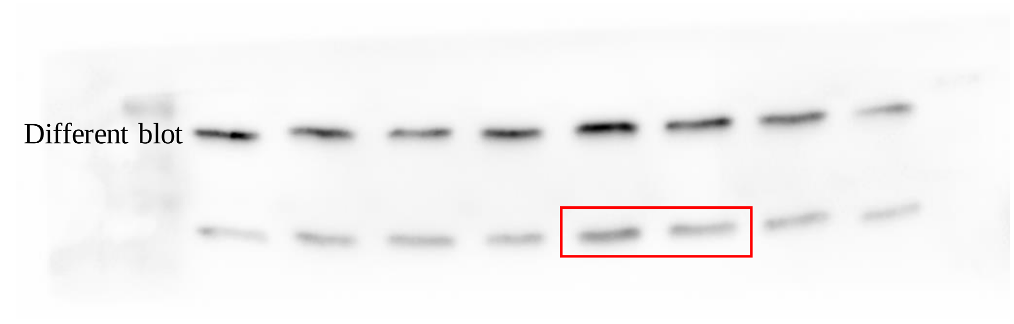

Fig. 6A
PGRMC1

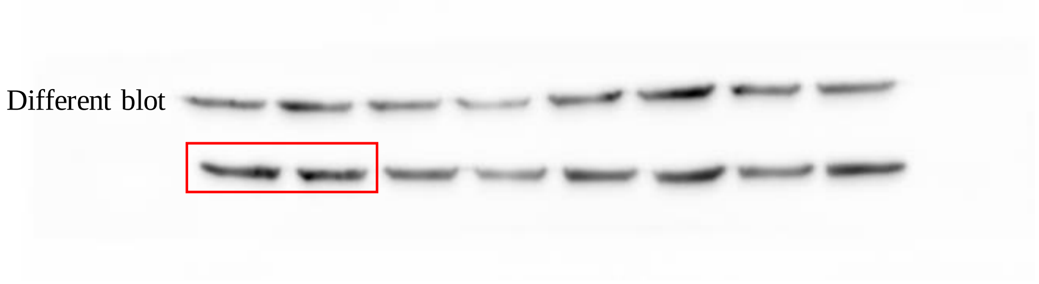

Fig. 6A
β-actin
